# Temperature and mental health–related emergency department and hospital encounters among children, adolescents and young adults

**DOI:** 10.1017/S2045796023000161

**Published:** 2023-04-17

**Authors:** Li Niu, Blean Girma, Bian Liu, Leah H. Schinasi, Jane E. Clougherty, Perry Sheffield

**Affiliations:** 1 Faculty of Psychology, Beijing Normal University, Beijing, China; 2 Department of Environmental Medicine and Public Health, Icahn School of Medicine at Mount Sinai, New York, NY, USA; 3 Department of Population Health Science and Policy, Icahn School of Medicine at Mount Sinai, New York, NY, USA; 4 Department of Environmental and Occupational Health and Urban Health Collaborative, Dornsife School of Public Health, Drexel University, Philadelphia, PA, USA

**Keywords:** climate change, extreme heat, paediatric, psychiatry, psychology

## Abstract

**Aims:**

We examine the association between high ambient temperature and acute mental health-related healthcare encounters in New York City for children, adolescents and young adults.

**Methods:**

This case-crossover study included emergency department (ED) visits and hospital encounters with a primary diagnosis of any mental health disorder during warm-season months (June–August) in New York City from 2005 to 2011 from patients of three age groups (6–11, 12–17 and 18–25 years). Using a distributed lag non-linear model over 0–5 lag days, by fitting a conditional logistic regression for each age group, we calculated the cumulative odds ratios of mental health encounters associated with an elevated temperature. Analyses were stratified by race/ethnicity, payment source and mental health categories to elucidate vulnerable subpopulations.

**Results:**

In New York City, there were 82,982 mental health–related encounters for young people aged 6 to 25 years during our study period months. Elevated temperature days were associated with higher risk of mental health–related ED and hospital encounters for the 6- to 11-year-olds (odds ratio [OR]: 1.28, 95% confidence interval [CI]: 1.13–1.46), for the 12- to 17-year-olds (OR: 1.17, 95% CI: 1.09–1.25) and for the 18- to 25-year-olds (OR: 1.09, 95% CI: 1.04–1.15). Children with reaction disorders, adolescents with anxiety and bipolar disorders, young adults with psychosis and reaction disorders and Black and non-Hispanic children and adolescents showed vulnerability to elevated temperature.

**Conclusions:**

We found that elevated ambient temperatures were associated with acute mental health ED or hospital encounters across childhood, adolescence and young adulthood.

## Introduction

Mental health problems among young people are a serious public health concern in the United States. One in six US children and adolescents between the ages of 6 and 17 years experiences a mental health disorder such as depression or anxiety problems (Whitney and Peterson, 2019). An estimated 30% of young adults aged 18 to 25 years meet the diagnostic criteria for a mental health disorder (Substance Abuse and Mental Health Services Administration, 2021). The mental health crisis in young people continues to grow in recent years. Adolescents and young adults experiencing serious mental health problems have increased by over 50% from the mid-2000s to 2017, and mental health–related emergency department (ED) visits by adolescents and young adults have increased by 56% from 2009 to 2015 (Santillanes *et al.*, 2020; Twenge *et al.*, 2019). The burden of mental health disorders on the healthcare system is significant, with 9%–12% of ED visits and nearly 10% of inpatient stays by young people were for a primary mental health diagnosis (Bardach *et al*., 2014; Santillanes *et al*., 2020).

A growing body of literature demonstrates links between ambient heat and acute mental health problems. Research shows that hospital and ED encounters for mental and behavioural disorders tend to increase during hot weather (Basu *et al*., 2018; Hansen *et al*., 2008; Nori-Sarma *et al*., 2022; Sherbakov *et al*., 2018). For example, Mullins and White (2019) reported a positive association of ambient temperature with multiple mental health outcomes among US individuals of all ages, including mental health–related ED visits, suicides and self-reported days of poor mental health. In a California study, Basu *et al*. (2018) found an increase in ED visits for mental health disorders associated with high temperatures during the warm season. Nori-Sarma *et al*. (2022) showed an association between heat and ED visits for mental health diagnoses for US adults in a national study. Positive associations between temperature and mental health–related healthcare encounters were reported in countries from different temperature zones, including Sweden, Australia, Canada, Vietnam, China and Korea (Carlsen *et al*., 2019; Chan *et al*., 2018; Hansen *et al*., 2008; Lee *et al*., 2018; Min *et al*., 2019; Peng *et al*., 2017; Trang *et al*., 2016; Wang *et al*., 2014). Previous research has also shown an association between higher ambient temperature and an increased risk of suicidal behaviour in multiple countries (Burke *et al*., 2018; Kim *et al*., 2019; Mullins and White, 2019; Sugg *et al*., 2019). There are several potential pathways by which heat can impact mental health illness. Exposure to ambient heat can exacerbate existing mental health conditions through altering thermosensitive physiological processes and causing negative mental states including distress and exhaustion (Noelke *et al*., 2016). Heat can also impact mental health through disrupted sleep, including difficulty falling asleep, poor sleep quality and fewer sleep hours (Mullins and White, 2019; Obradovich *et al*., 2017). Heat exposure has also been shown to increase irritability, aggression and violence, which are associated with adverse mental health outcomes (Anderson, 2001).

The existing literature on links between ambient temperature and mental health has focused on the adult population; however, little is known about heat impacts on mental health across childhood, adolescence and young adulthood, which are key developmental periods with special risk for mental health disorders (Qiu *et al*., 2022). Children and adolescents are less able to regulate their emotions and behaviours, are more prone to impulsive behaviours and are less likely to take adaptive measures such as avoidance of high-intensity physical activities compared to adults, which can increase the risk for mental health illness during periods of high temperatures (Santillanes *et al*., 2020). Young adults experience high emotional burden from social, school and work responsibilities, and many face economic and social barriers that further contribute to mental health challenges (Rahal *et al*., 2020). Only a few studies have examined associations between ambient temperature and acute mental health outcomes in young people, and those few have reported mixed results (Basu *et al*., 2018; Chan *et al*., 2018; Trang *et al*., 2016; Wang *et al*., 2014).

This study examines associations between high ambient temperature and risk of mental health–related healthcare encounters (i.e., ED visits and hospitalization) among young people aged 6 to 25 years living in New York City (NYC). We hypothesized that all three age groups (children, 6–11 years; adolescents, 12–17 years young adults, 18–25 years) would be at increased risk for heat impact on mental health. We further identify subgroups of young people who are particularly vulnerable to heat by stratifying by sex, race/ethnicity, payment source and mental health diagnostic groups. Based on previous work that heat increases emotional distress and induces impulsiveness and aggression (Anderson, 1989; Kim *et al*., 2016), we expected that emotional (e.g., depression and anxiety) and behavioural disorders (e.g., externalizing) would be more strongly impacted by heat than other mental health categories. Given that children and adolescents from racially marginalized groups and low socio-economic positions are disproportionately impacted by heat and often lack resources (e.g., air conditioning) to cope with heat (Gronlund, 2014; Renteria *et al*., 2022), we expected that racial/ethnic minority groups and those paying with Medicaid (indicating low income) would be more vulnerable to high temperature in terms of mental health risk. Due to mixed findings related to sex (Basu *et al*., 2018; Kim *et al*., 2019; Nori-Sarma *et al*., 2022), no specific hypothesis was made for how sex would moderate the association between temperature and mental health risk.

## Methods

### Health data and study population

Data on all daily ED visits and hospitalizations in NYC for the period from 2005 to 2011 were extracted from the New York Statewide Planning and Research Cooperative System (SPARCS). SPARCS is a comprehensive all-payer administrative claims database for inpatient stays and outpatient (ambulatory surgery, ED and outpatient services) visits in New York State (New York State Department of Health, 2015). It includes patient-level information on demographic characteristics, diagnoses and treatments, payment sources and charges for each hospital discharge record. We restricted our analysis to ED visits and hospitalizations that occurred during the warm-season months of June, July and August between 2005 and 2011, as we sought to study the heat effects on mental health–related encounters (Sheffield *et al*., 2018). Because of the relative acute nature of the hypothesized heat effect on mental health, we excluded scheduled inpatient visits (e.g., elective surgery) as well as inpatient and outpatient visits that resulted from circumstances other than a disease or injury (e.g., routine child health check). We focused on ED visits and hospitalizations for young people between the ages of 6 and 25 years to capture developmental stages when mental health disorders emerge, increase and peak (Bitsko, 2022).

### Health outcomes

Mental health–related ED and hospital encounters were identified using each patient’s primary diagnosis coded with the International Classification of Diseases, Ninth Revision (ICD-9). The primary outcome of interest is the presence of any primary diagnosis indicative of mental health disorders (ICD-9 codes 290–299), following several prior studies studying acute weather impacts on mental health encounters (Basu *et al*., 2018; Chan *et al*., 2018; Sherbakov *et al*., 2018). We defined our outcome as those with mental health disorders as the primary diagnosis and excluded cases with another cause as the primary diagnosis and a mental health diagnosis as the secondary diagnosis (e.g., patients with existing mental health disorders who become ill with other causes during hot weather). We grouped the diagnoses into 17 subcategories of mental health diagnosis using the definitions developed for the paediatric patient population by Bardach *et al*. (2014): anxiety disorders, bipolar disorder, depression, externalizing disorders (e.g., conduct and oppositional defiant disorders), psychosis, reaction disorders (i.e., an unhealthy or excessive emotional or behavioural response to a stressful event or change in a person’s life), substance use disorders, attention–deficit/hyperactivity disorder (ADHD), attachment, autism, developmental, eating, elimination, motor, personality, sexuality and other mental health disorders. We also coded a separate category for suicide and self-inflicted injury if an encounter had an associated external cause of injury code identified as self-inflicted injury/suicide (ICD-9 codes E95). The ICD-9 codes used for the identification of mental health diagnostic categories were included as Supplementary Table S1.

We also extracted from SPARCS information about patients’ race/ethnicity (mutually exclusive categories of Hispanic, non-Hispanic Black, non-Hispanic White or non-Hispanic other) and payment sources based on the primary source of payment (coded as mutually exclusive categories of commercial, Medicaid, self-pay or other payment sources including worker’s compensation, Medicare, other federal programme, blue cross, CHAMPUS (a Department of Defense health care program now called TRICARE) and other non-federal programme).

### Climate exposure and meteorological data

We obtained data on daily temperatures from the four NYC-area meteorological stations (Central Park, JFK International Airport, LaGuardia Airport and Newark International Airport) from the National Oceanic and Atmospheric Administration (NOAA) National Climate Data Center (NCDC) (NOAA National Centers for Environmental Information, 2020). The data from the four stations were averaged to create a citywide daily trend. Because disruption of sleep is a critical mechanism through which temperature may affect mental health episodes (Obradovich *et al*., 2017), we used the daily minimum temperature (*T*_min_) – a strong indicator of nighttime temperature – as our main exposure variable (Barnett *et al*., 2010; Sheffield *et al*., 2018; Winquist *et al*., 2016). We found *T*_min_ to be highly correlated with the daily maximum temperature (*r* = 0.96) and mean temperature (*r* = 0.99). We calculated relative humidity (RH) from mean temperature and dew point temperature following the standard NOAA equation using the R package *weathermetrics* (Anderson *et al*., 2016).

### Statistical analysis

We used a case-crossover approach to study associations between daily *T*_min_ and mental health–related ED and hospital visits (Maclure, 1991). For each subject who had an ED or hospital visit, we used the date of the visit as the case day and then matched control days to each case day based on day of the week, month and year, resulting in 3 or 4 control days per case day (Janes *et al*., 2005). Because cases serve as their own controls, this approach adjusts for individual time-independent factors (Maclure, 1991). By matching the case day to all of the same days of week within the same month, it also controls for long-term time trends, seasonality and day of week (Schinasi *et al*., 2020).

We then performed conditional logistic regression to estimate the effects of temperature on the risk of mental health–related ED visits and hospitalizations (Buteau *et al*., 2018). We modelled the temperature variable using the distributed lag non-linear model (DLNM) function, a modelling framework that allows, simultaneously, for non-linear and lagged effects in exposure–outcome associations. We modelled the effect of *T*_min_ over lag days 0 to 5. We selected a lag period of 6 days based on prior research showing that the heat effects on mental health are relatively acute, with the most adverse effects of heat apparent within several days (Lee *et al*., 2018; Min *et al*., 2019; Wang *et al*., 2014). In keeping with previous practice, we modelled *T*_min_ as a natural cubic spline of 3 degrees of freedom (df) with integer lag functions. Exploratory analyses using an alternative 5 df produced a similar pattern in exposure–response associations (Supplementary Appendix A). As such, we chose 3 df because it was parsimonious and provided a reliable fit to the data.

For all models, we estimated the cumulative odds ratios (ORs) over lags 0 to 5 for a temperature increment from the minimum risk temperature (MRT) to an elevated temperature, defined as the 95th percentile of distribution of the warm-season (June–August) temperature from 2005 to 2011. The MRT were defined as the *T*_min_ at which the least mental health–related encounters were observed. We used it as the reference point for calculating the OR to allow flexibility in interpreting heat impacts for different age groups (e.g., the optimal temperature for children may be different in adolescents or young adults).

We examined the association between *T*_min_ and mental health–related disorders separately for three age groups: 6–11 years (children), 12–17 years (adolescence) and 18–25 years (young adults). These stratified analyses were conducted because childhood, adolescence and young adulthood represent development stages with distinct healthcare utilization patterns, contributing factors and underlying mechanisms associated with mental health and heat vulnerability. We combined mental health–related ED visits and hospital encounters in the main analysis because an investigation of ED and hospitalizations as separate outcomes yielded similar shapes of temperature–outcome associations in each age group (Supplementary Appendix B).

To identify subgroups of individuals at heightened risk for mental health episodes associated with heat, we examined temperature–outcome associations by sex, race/ethnicity, payment source and mental health diagnostic categories separately for children, adolescents and young adults. Subgroups with small sample sizes (i.e., less than 500) were not examined due to insufficient power.

As sensitivity analyses, we tested whether including RH as a covariate would change the temperature–outcome associations. We also fitted parallel models limiting to cases during July and August to examine the possible influence of the school year (e.g., the public school is closed from June to late August).

All statistical analyses were conducted using the R statistical software version 4.0.2 (R Core Team, 2015) with the ‘*dlnm*’ and ‘*coxph*’ packages (Gasparrini, 2011). Institutional review boards at the authors’ institutions approved this study.

## Results

As shown in Table 1, during the period from June to August between 2005 and 2011 in NYC, there were 82,982 mental health–related healthcare encounters (59,731 ED visits and 23,251 hospitalizations) among children (*n* = 6,268), adolescents (*n* = 22,206) and young adults (*n* = 54,508). Females comprised 44% of the healthcare encounters (32% for 6–11 years, 53% for 12–17 years and 41% for 18–25 years). Non-Hispanic Black (35%) individuals represented the largest group, followed by Hispanic (24%), non-Hispanic other (23%) and non-Hispanic White individuals (18%). Nearly a third of the encounters were paid by commercial insurance (31%); Medicaid accounted for 25% and self-pay accounted for 19% of the encounters. The most common mental health diagnoses were externalizing disorders for children aged 6 to 11 years (27%), depression for adolescents aged 12 to 17 years (21%) and substance use disorders for young adults aged 18 to 25 years (27%). Descriptive statistics of mental health–related encounters, separate for hospitals and EDs, are provided in Supplementary Table S2. The range of *T*_min_ over the study period was 50–83°F, with a mean of 67.9°F and a standard deviation of 5.4°F (see details of exposure data in Supplementary Table S3).

**Table 1.** Descriptive statistics of mental health–related ED and hospital admissions in NYC during the warm season (June–August), 2005–2011

Characteristics	Age 6−11 years (*n* = 6,268)	Age 12−17 years (*n* = 22,206)	Age 18−25 years (*n* = 54,508)	Overall (*n* = 82,982)
Sex, *n* (%)				
Female	1,993 (31.8)	11,868 (53.4)	22,306 (40.9)	36,167 (43.6)
Male	4,275 (68.2)	10,337 (46.6)	32,201 (59.1)	46,813 (56.4)
Race/ethnicity, *n* (%)				
Hispanic	1,634 (26.1)	5,780 (26.0)	12,216 (22.4)	19,630 (23.7)
Non-Hispanic Black	2,864 (45.7)	8,257 (37.2)	17,687 (32.4)	28,808 (34.7)
Non-Hispanic other	1,173 (18.7)	5,037 (22.7)	12,912 (23.7%)	19,122 (23.0)
Non-Hispanic White	540 (8.6)	3,001 (13.5)	11,374 (20.9)	14,915 (18.0)
Unknown/Unreported	57 (0.9)	131 (0.6)	319 (0.6)	507 (0.6)
Payment source, *n* (%)				
Commercial	2,286 (36.5)	8,774 (39.5)	14,415 (26.4)	25,475 (30.7)
Medicaid	1,827 (29.1)	4,766 (21.5)	14,049 (25.8)	20,642 (24.9)
Self-pay	598 (9.5)	2,676 (12.1)	12,580 (23.1)	15,854 (19.1)
Others	221 (3.5)	1,314 (5.9)	3,512 (6.4)	5,047 (6.1)
Unknown/Unreported	1,336 (21.3)	4,676 (21.1)	9,952 (18.3)	15,964 (19.2)
Mental health diagnosis, *n* (%)				
Anxiety disorders	532 (8.5)	1,884 (8.5)	5,822 (10.7)	8,238 (9.9)
Bipolar disorder	201 (3.2)	1,749 (7.9)	4,853 (8.9)	6,803 (8.2)
Depression	770 (12.3)	4,632 (20.9)	7,658 (14.0)	13,060 (15.7)
Externalizing disorders	1,684 (26.9)	4,561 (20.5)	1,451 (2.7)	7,696 (9.3)
Psychosis	275 (4.4)	1,477 (6.7)	11,990 (22.0)	13,742 (16.6)
Reaction disorders	600 (9.6)	1,786 (8.0)	3,189 (5.9)	5,575 (6.7)
Substance abuse	17 (0.3)	2,385 (10.7)	14,505 (26.6)	16,907 (20.4)
Suicide/self-inflicted injury	61 (1.0)	1,279 (5.8)	2,961 (5.4)	4,301 (5.2)
Others	2,128 (34.0)	2,453 (11.0)	2,079 (3.8)	6,660 (8.0)

*Notes*: Externalizing disorders include conduct and oppositional defiant disorders. Other mental health diagnosis category includes ADHD, attachment, autism, developmental, eating disorders, elimination, others, motor, personality, sexuality and other mental health disorders. Other payment sources include worker’s compensation, Medicare, blue cross, CHAMPUS, other federal programme and other non-federal programme.

### Associations between temperature and mental health–related healthcare encounters

**Figure 1.**
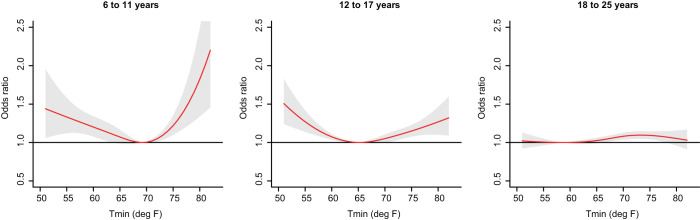
Cumulative odds ratio and 95% confidence intervals of mental health–related ED and hospital admissions associated with daily minimum temperature over lag 0–5 days, June to August.

The shape of the exposure–outcome associations differed by age groups (Figure 1). Among children, we observed a ‘U’-shaped relationship between daily minimum temperature and risk of mental health–related encounters, with the lowest risk at 69.2°F (i.e., MRT). Compared to the MRT, an elevated temperature (76°F, 95th percentile) was associated with higher odds of mental health events (OR: 1.28, 95% confidence interval [CI]: 1.13–1.46) accumulative over lag 0 to 5 days. Among adolescents, we observed a similar, yet attenuated, ‘U’ shape, with a slightly cooler MRT (65.2°F); the OR comparing an elevated temperature (76°F, 95th percentile) to MRT was 1.17 (95% CI: 1.09–1.25). For young adults, the exposure–outcome curve was almost flat, with a small increase in risk of mental health event from around 65°F to 72°F, which then levelled off with increasing temperatures. The OR comparing an elevated temperature (76°F, 95th percentile) with the MRT (58.7°F) was 1.09 (95% CI: 1.04–1.15).

### Stratification by sex, race/ethnicity, payment sources and mental health diagnostic categories

In children, we identified several subgroups at significant risk for mental health encounters associated with an elevated temperature (95th percentile compared to the MRT). They included children from non-Hispanic Black (OR: 1.43, 95% CI: 1.12–1.83) or other racial/ethnic groups (OR: 1.60, 95% CI: 1.12–2.27) and those paid with Medicaid (OR: 1.48, 95% CI: 1.09–2.02). Both males (OR: 1.28, 95% CI: 1.09–1.52) and females (OR: 1.34, 95% CI: 1.06–1.71) were at risk for mental health encounters at an elevated temperature. Among all mental health diagnostic groups examined, the strongest associations were found among children diagnosed with reaction disorders (OR: 2.21, 95% CI: 1.22–3.99) (Table 2).

**Table 2.** Cumulative ORs and 95% CIs of mental health–related ED and hospital admissions associated with elevated minimum temperature (95th percentile) relative to the MRT, overall and by sex, race, payment source and mental health diagnosis subgroups

	6−11 years		12−17 years		18−25 years	
	Minimum risk temperature	OR (95% CI)	Minimum risk temperature	OR (95% CI)	Minimum risk temperature	OR (95% CI)
All admissions	69.2	*1.28 (1.13, 1.46)*	65.2	*1.17 (1.09, 1.25)*	58.7	*1.09 (1.04, 1.15)*
Sex						
Female	*68.2*	*1.34 (1.06, 1.71)*	*64.4*	*1.26 (1.14, 1.39)*	54.8	1.12 (0.99, 1.27)
Male	*69.5*	*1.28 (1.09, 1.52)*	*69.0*	*1.11 (1.00, 1.22)*	*60.3*	*1.07 (1.00, 1.14)*
Race						
Hispanic	63.3	1.37 (0.99, 1.90)	66.4	1.14 (0.97, 1.33)	50.5	1.17 (0.87, 1.58)
Non-Hispanic Black	69.9	*1.43 (1.12, 1.83)*	64.1	*1.21 (1.06, 1.39)*	50.5	1.13 (0.88, 1.44)
Non-Hispanic White	70.6	1.33 (0.78, 2.28)	66.7	1.19 (0.97, 1.47)	82.7	1.20 (0.97, 1.49)
Non-Hispanic Other	69.7	*1.60 (1.12, 2.27)*	68.6	*1.26 (1.07, 1.49)*	62.3	*1.12 (1.01, 1.25)*
Payment source						
Commercial	70.2	1.19 (0.88, 1.60)	64.3	*1.25 (1.08, 1.44)*	50.5	1.29 (0.98, 1.71)
Medicaid	66.9	*1.48 (1.09, 2.02)*	63.8	1.11 (0.92, 1.34)	62.8	*1.20 (1.08, 1.33)*
Self-pay	70.4	1.71 (0.90, 3.27)	63.8	1.21 (0.91, 1.60)	82.7	*1.33 (1.06, 1.67)*
Others	–	–	71.4	1.15 (0.81, 1.63)	50.5	1.43 (0.80, 2.56)
Mental health diagnosis						
Anxiety disorders	50.5	1.66 (0.45, 6.12)	63.1	*1.42 (1.08, 1.87)*	82.7	1.15 (0.85, 1.57)
Bipolar disorder	–	–	69.6	*1.39 (1.06, 1.83)*	50.5	1.37 (0.85, 2.22)
Depression	69.5	1.31 (0.83, 2.07)	66.8	1.13 (0.94, 1.36)	82.7	1.26 (0.96, 1.66)
Externalizing disorders	69.0	1.20 (0.86, 1.68)	65.6	1.08 (0.90, 1.30)	50.5	1.97 (0.83, 4.68)
Psychosis	–	–	57.5	1.34 (0.93, 1.94)	65.1	*1.14 (1.03, 1.27)*
Reaction disorders	69.7	*2.21 (1.22, 3.99)*	63.8	1.23 (0.90, 1.68)	58.5	*1.32 (1.04, 1.67)*
Substance abuse	–	–	82.7	1.06 (0.66, 1.72)	50.5	1.20 (0.91, 1.60)
Suicide/self-inflicted injury	–	–	65.8	1.19 (0.85, 1.67)	50.5	1.61 (0.87, 2.98)

We did not present results for small subgroups (less than 500) due to large margins of error. Italic estimates indicate significant associations with a *P*-value greater than 0.05

In adolescents, we identified subgroups at significant risk for mental health encounters associated with an elevated temperature (95th percentile) compared to the MRT, including adolescents from non-Hispanic Black (OR: 1.21, 95% CI: 1.06–1.39) or other racial/ethnic groups (OR: 1.26, 95% CI: 1.07–1.49) and those who paid with commercial insurance (OR: 1.25, 95% CI: 1.08–1.44). Both male (OR: 1.11, 95% CI: 1.00–1.22) and female adolescents (OR: 1.26, 95% CI: 1.14–1.39) were at risk for mental health encounters at an elevated temperature. Adolescents diagnosed with anxiety (OR: 1.42, 95% CI: 1.08–1.87) or bipolar disorders (OR: 1.39, 95% CI: 1.06–1.83) showed significant vulnerability to elevated temperature, among all diagnostic categories (Table 2).

For young adults, elevated temperatures (95th percentile compared to the MRT) conferred significant risk for mental health encounters among several subgroups, including young adults who were male (OR: 1.07, 95% CI: 1.00–1.14), those from other racial/ethnic groups (OR: 1.12, 95% CI: 1.01–1.25), those who paid with Medicaid (OR: 1.20, 95% CI: 1.08–1.33) or self-paid (OR: 1.33, 95% CI: 1.06–1.67), as well as those diagnosed with psychosis (OR: 1.14, 95% CI: 1.03–1.27) or reaction disorders (OR: 1.32, 95% CI: 1.04–1.67) (Table 2).

The shape of exposure–outcome associations for all subgroup analyses is shown in Supplementary Figures S3–S6. Within each of the three age groups, we performed heterogeneity test to assess significant differences in ORs by sex, race, payment source and mental health diagnosis group (Kaufman and MacLehose, 2013). We did not find statistical evidence for heterogeneous effect by these factors, as indicated by the Cochran’s *Q* test statistic (all *P* > 0.05, Supplementary Table S8).

### Sensitivity analysis

We found that temperature–outcome associations were robust to adjustment for RH (Supplementary Appendix C), with very similar results for all age groups (children, OR: 1.28, 95% CI: 1.13–1.46; adolescents, OR: 1.17, 95% CI: 1.09–1.26; young adults, OR: 1.09, 95% CI: 1.04–1.15). Restricting analyses to only July and August produced stronger effects of elevated temperatures on mental health–related encounters (children, OR: 1.44, 95% CI: 1.20–1.74; adolescents, OR: 1.62, 95% CI: 1.27–2.08; young adults, OR: 1.29, 95% CI: 1.11–1.50) (Supplementary Appendix D).

## Discussion

We found associations between daily minimum temperature and mental health–related healthcare encounters among children, adolescents and young adults in a large urban setting. Associations were strongest among children and adolescents, though still elevated in young adults. We also identified subgroups of young people at heightened risk for mental health encounters associated with heat. Our findings contribute to the understanding of the complex links between heat exposure and mental health among young people. As extreme temperatures become more frequent and severe with global climate change, findings from this study can help inform targeted prevention strategies to improve mental health during periods of extreme heat in different youth populations.

Prior studies have found mixed results on whether children and adolescents have higher risk for mental health encounters in association with high ambient heat. For example, stronger heat–outcome associations were observed among individuals aged 6 to 18 years in Basu *et al*. (2018) and individuals aged 0 to 14 years in Wang *et al*. (2014) compared to older age groups (Basu *et al*., 2018; Wang *et al*., 2014), whereas two other studies have found that young people are less impacted by heat than are adults or elderly persons (Chan *et al*., 2018; Trang *et al*., 2016). Our study provided additional evidence that the impact of heat on acute mental health encounters were strongest among children and adolescents, though still elevated in young adults. The younger age groups were more affected by heat than young adults likely because of age-related physiological vulnerability (e.g., younger children are less able to regulate core body temperature). In addition, young adults may have better ability to protect themselves in terms of avoiding heat exposure (e.g., staying indoor and turning on air conditioning) and taking early-stage interventions to prevent severe mental healthcare outcomes (e.g., seeking social support and taking treatments and consulting therapists) and also possibly more hours per day exposure to air conditioning in workplace settings than children and adolescents (Sugg *et al*., 2019). Several common mechanisms could underlie the vulnerability to high temperature in terms of mental health risk among the three age groups. These include sleep disturbance, heat stress and/or dehydration contributing to altered neuroendocrine chemistry and also social aggression mechanisms that contribute to conflict and violence (Manchia *et al*., 2017; Tiihonen *et al*., 2017). Also, individuals with mental health conditions can have impaired cognitive awareness that make them less able to cope with heat, resulting in an exacerbation of mental health episodes.

The types of mental health diagnoses most strongly associated with temperature differed by age group: reaction disorder for children, anxiety and bipolar disorders among adolescents and psychosis and reaction disorders for young adults. Our findings for young adults were consistent with a recent national study of adults that also found associations between heat and schizotypal and anxiety disorders, which had overlapping diagnostic codes with our psychosis and reaction disorder categories (Nori-Sarma *et al*., 2022). They also found significant positive associations between ambient heat and hospitalizations related to substance use disorders, where we observed positive but non-significant associations possibly due to our smaller sample size (Nori-Sarma *et al*., 2022). Other studies have also shown increases in alcohol consumption and drug abuse during heatwaves (Cusack *et al*., 2011). In contrast to previous studies that have reported a positive association between temperature and suicide (Burke *et al*., 2018; Kim *et al*., 2019; Mullins and White, 2019; Sugg *et al*., 2019), we find essentially no evidence of an association between heat exposure and suicidal behaviour. It is likely that our small number of hospital and ED encounters identified with suicide or self-inflicted injury diagnostic codes led to imprecise and unstable effect estimates. Future research with large data sets and in different locations could help to clarify developmental changes in heat vulnerability related to mental health problems and suicide and shed light on risk factors and mechanisms.

We also found significant positive associations between heat and mental health–related encounters among children and adolescents who were non-Hispanic Black or from other racial/ethnic groups. Our results suggest that racially marginalized youth, a population disproportionately impacted by micro-urban heat islands effects, may need targeted prevention efforts during heatwaves. We found that payment sources with the highest heat vulnerability differed by age group. Although heat conferred risk for mental health encounters paid with Medicaid among children, heat risk was found for those who self-paid among adolescents, perhaps indicating the complicated and often inconsistent health insurance coverage particularly experienced by young people from low-income backgrounds.

Similar to a few previous studies (Kim *et al*., 2019; Sugg *et al*., 2019), our study has shown a ‘U’-shaped relationship between temperature and mental health ED and hospital encounters among children and adolescents, which suggests that the risk of mental health encounters may increase at both high and low temperature extremes. We found that the MRT differed across age groups and across racial/ethnic, payment source and mental health diagnostic subgroups. MRT indicates the temperature at which the lowest risk of mental health–related encounters is observed. While not well-studied, the MRT variation we observed complements our findings of variations in the magnitude of association with high temperature and mental health outcomes. It also suggests that behavioural and potentially physiologic susceptibility to heat may vary by age and social factors and depend on the type of mental health outcome.

This study has a number of limitations. First, our study examined associations, not causal effects, of heat and mental health. Second, the study is not generalizable beyond large US cities, as it includes data only from NYC health facilities, though there are some consistencies with results from other states or nationwide data (Mullins and White, 2019; Nori-Sarma *et al*., 2022; Palinkas and Wong, 2020). Third, we were unable to control for potential biases in mental health diagnostic patterns (Bailey *et al*., 2019; Fadus *et al*., 2020; Snowden, 2003), particularly those across racial or socio-economic subgroups. Fourth, although the study design of a case-crossover analysis helps us remove many of the long-term psychosocial, climate or other factors that may confound the associations between temperature and mental health, there are weather co-exposures (e.g., daily precipitation level) and air pollution exposures that have a potential to correlate with both the temperature level and mental health risk. Future research needs to consider how temperatures, in conjunction with weather and air pollution co-exposures, contribute to increased mental health issues in young people. Fifth, while many of the participants presenting at ED or being hospitalized for mental disorders have pre-existing mental health problems, we do not have data on participants’ pre-existing mental health conditions. Previous research has shown that the use of psychotropic medications can disrupt normal thermoregulation, and children and adolescents may experience more severe reactions to psychotropic medications (Cooper *et al*., 2014; Stöllberger *et al*., 2009). Future research needs to elucidate the role of pre-existing mental health conditions and use of medications on increasing mental health risk associated with heat. Further, this study does not have data on sleep patterns, which suggests caution in making interpretations about the impact of high temperatures on sleep disturbance as a potential pathway to mental health disorders. Finally, our study did not examine the effect of heat during the cold season and our data are from 2005 to 2011 and thus do not capture the present mental health crises precipitated by the COVID-19 pandemic of 2020 and beyond. To address these limitations and other research gaps, future studies will hopefully include larger sample sizes and investigate broader exposures, including indoor temperatures, to better understand the sleep disruption mechanism and the impact of multiple extreme weather events on mental health.

In summary, we observed increased healthcare utilization for multiple mental health diagnoses associated with high temperatures across childhood, adolescence and young adulthood. As ED and hospital encounters represent the more severe cases of mental health crises, these findings may underestimate the true toll of heat on mental health. As sleep disturbance is a hypothesized mechanism, future studies could examine indoor temperature and the potential protective role well-functioning air conditioning may play, particularly for those communities disproportionately burdened by micro-urban heat islands.

## Data Availability

Data are available from New York State Department of Health by application and signed data user agreement.

## Supplementary material

For supplementary material accompanying this paper visit http://doi.org/10.1017/S2045796023000161. click here to view supplementary material
