# Supplementary material for: Temperature and mental health–related emergency department and hospital encounters among children, adolescents and young adults
Source: Epidemiol Psychiatr Sci. 2023 Apr 17;32:e22. doi: 10.1017/S2045796023000161 (PMC10130844; doi:10.1017/S2045796023000161)
Supplement: Supplementary file 1 [file S2045796023000161sup001.docx]

**Supplementary Materials**

**Table of Contents**

Table S1. ICD-9 Code Definitions for mental health diagnostic groupings.

Table S2. Descriptive statistics of mental health-related admissions, separate for hospitals and emergency departments (ED) in New York City, July to August, 2005-2011.

Table S3 Distribution and correlation of selected weather variables for all years, June to August, and July to August, 2005-2011, New York City.

Appendix 1. Model results using natural cubic spline with 5 degrees of freedom.

Figure S1. Cumulative odds ratio (ORs) and 95% confidence intervals of mental health-related emergency department and hospital admissions associated with daily minimum temperature over lag 0-5 days, June to August, using natural cubic spline with 5 degrees of freedom.

Table S4. Cumulative odds ratio (ORs) and 95% confidence intervals of mental health-related emergency department and hospital admissions associated with elevated minimum temperature (95^th^ percentile) relative to the minimum risk temperature over lag 0-5 days, June to August, using natural cubic spline with 5 degrees of freedom.

Appendix 2. Model results for separate hospital and emergency departments (ED) admission outcomes.

Figure S2. Cumulative odds ratio (ORs) and 95% confidence intervals of mental health-related admissions, separate for hospitals and emergency departments (ED), associated with daily minimum temperature over lag 0-5 days, June to August.

Table S5. Cumulative odds ratio (ORs) and 95% confidence intervals of mental health-related admissions, separate for hospitals and emergency departments (ED), associated with elevated minimum temperature (95^th^ percentile) relative to the minimum risk temperature.

Figure S3. Cumulative odds ratio (ORs) and 95% confidence intervals of mental health-related emergency department and hospital admissions associated with daily minimum temperature over lag 0-5 days, June to August, for sex subgroups.

Figure S4. Cumulative odds ratio (ORs) and 95% confidence intervals of mental health-related emergency department and hospital admissions associated with daily minimum temperature over lag 0-5 days, June to August, for race subgroups.

Figure S5. Cumulative odds ratio (ORs) and 95% confidence intervals of mental health-related emergency department and hospital admissions associated with daily minimum temperature over lag 0-5 days, June to August, for payment sources subgroups.

Figure S6. Cumulative odds ratio (ORs) and 95% confidence intervals of mental health-related emergency department and hospital admissions associated with daily minimum temperature over lag 0-5 days, June to August, for mental health diagnostic subgroups.

Appendix 3. Model results including relative humidity as a covariate.

Figure S7. Cumulative odds ratio (ORs) and 95% confidence intervals of mental health-related emergency department and hospital admissions associated with daily minimum temperature over lag 0-5 days, June to August, adjusting for relative humidity.

Table S6. Cumulative odds ratio (ORs) and 95% confidence intervals of mental health-related emergency department and hospital admissions associated with elevated minimum temperature (95^th^ percentile) relative to the minimum risk temperature over lag 0-5 days, June to August, adjusting for relative humidity.

Appendix 4. Model results limiting to only July and August.

Figure S8. Cumulative odds ratio (ORs) and 95% confidence intervals of mental health-related emergency department and hospital admissions associated with daily minimum temperature over lag 0-5 days, July and August.

Table S7 Cumulative odds ratio (ORs) and 95% confidence intervals of mental health-related emergency department and hospital admissions associated with elevated minimum temperature (95^th^ percentile) relative to the minimum risk temperature over lag 0-5 days, July and August.

Table S8. Heterogeneity test of odds ratio (ORs) of the association between mental health-related emergency department and hospital admissions with elevated minimum temperature (95^th^ percentile) relative to the minimum risk temperature (MRT), by sex, race, payment sources, and mental health diagnosis subgroups.

Table S1. ICD-9 Code Definitions for mental health diagnostic categories.

| Mental health diagnostic categories | ICD-9 Code | Definition |
| --- | --- | --- |
| Anxiety | 300-300.3, 300.5-300.9, 309.21,  309.81 | [300](http://www.icd9data.com/2015/Volume1/290-319/300-316/300/300.htm) Anxiety, dissociative and somatoform disorders  [300.0](http://www.icd9data.com/2015/Volume1/290-319/300-316/300/300.0.htm) Anxiety states  [300.1](http://www.icd9data.com/2015/Volume1/290-319/300-316/300/300.1.htm) Dissociative, conversion and factitious disorders  [300.2](http://www.icd9data.com/2015/Volume1/290-319/300-316/300/300.2.htm) Phobic disorders  [300.3](http://www.icd9data.com/2015/Volume1/290-319/300-316/300/300.3.htm) Obsessive-compulsive disorders  [300.5](http://www.icd9data.com/2015/Volume1/290-319/300-316/300/300.5.htm) Neurasthenia  [300.6](http://www.icd9data.com/2015/Volume1/290-319/300-316/300/300.6.htm) Depersonalization disorder  [300.7](http://www.icd9data.com/2015/Volume1/290-319/300-316/300/300.7.htm) Hypochondriasis  [300.8](http://www.icd9data.com/2015/Volume1/290-319/300-316/300/300.8.htm) Somatoform disorders  [300.9](http://www.icd9data.com/2015/Volume1/290-319/300-316/300/300.9.htm) Unspecified nonpsychotic mental disorder  [309.21](http://www.icd9data.com/2015/Volume1/290-319/300-316/309/309.21.htm) Separation anxiety disorder  [309.81](http://www.icd9data.com/2015/Volume1/290-319/300-316/309/309.81.htm) Posttraumatic stress disorder |
| Bipolar | 296-296.16, 296.4-296.89 | [296](http://www.icd9data.com/2015/Volume1/290-319/295-299/296/296.htm) Episodic mood disorders  [296.0](http://www.icd9data.com/2015/Volume1/290-319/295-299/296/296.0.htm) Bipolar i disorder, single manic episode  [296.1](http://www.icd9data.com/2015/Volume1/290-319/295-299/296/296.1.htm) Manic disorder recurrent episode  [296.4](http://www.icd9data.com/2015/Volume1/290-319/295-299/296/296.4.htm) Bipolar i disorder, most recent episode (or current) manic  [296.5](http://www.icd9data.com/2015/Volume1/290-319/295-299/296/296.5.htm) Bipolar i disorder, most recent episode (or current) depressed  [296.6](http://www.icd9data.com/2015/Volume1/290-319/295-299/296/296.6.htm) Bipolar i disorder, most recent episode (or current) mixed  [296.7](http://www.icd9data.com/2015/Volume1/290-319/295-299/296/296.7.htm) Bipolar I disorder, most recent episode (or current) unspecified  [296.8](http://www.icd9data.com/2015/Volume1/290-319/295-299/296/296.8.htm) Other and unspecified bipolar disorders |
| Depression | 296.2-296.39, 311,  300.4,  296.9-296.99 | [296.2](http://www.icd9data.com/2015/Volume1/290-319/295-299/296/296.2.htm) Major depressive disorder single episode  [296.3](http://www.icd9data.com/2015/Volume1/290-319/295-299/296/296.3.htm) Major depressive disorder recurrent episode  [311](http://www.icd9data.com/2015/Volume1/290-319/300-316/311/311.htm) Depressive disorder, not elsewhere classified  [300.4](http://www.icd9data.com/2015/Volume1/290-319/300-316/300/300.4.htm) Dysthymic disorder  [296.9](http://www.icd9data.com/2015/Volume1/290-319/295-299/296/296.9.htm) Other and unspecified episodic mood disorder |
| Externalizing | 312-313.82 | [312](http://www.icd9data.com/2015/Volume1/290-319/300-316/312/312.htm) Disturbance of conduct not elsewhere classified  [313](http://www.icd9data.com/2015/Volume1/290-319/300-316/313/313.htm) Disturbance of emotions specific to childhood and adolescence  [313.0](http://www.icd9data.com/2015/Volume1/290-319/300-316/313/313.0.htm) Overanxious disorder specific to childhood and adolescence  [313.1](http://www.icd9data.com/2015/Volume1/290-319/300-316/313/313.1.htm) Misery and unhappiness disorder specific to childhood and adolescence  [313.2](http://www.icd9data.com/2015/Volume1/290-319/300-316/313/313.2.htm) Sensitivity shyness and social withdrawal disorder specific to childhood and adolescence  [313.3](http://www.icd9data.com/2015/Volume1/290-319/300-316/313/313.3.htm) Relationship problems specific to childhood and adolescence  [313.8](http://www.icd9data.com/2015/Volume1/290-319/300-316/313/313.8.htm) Other or mixed emotional disturbances of childhood or adolescence  [313.81](http://www.icd9data.com/2015/Volume1/290-319/300-316/313/313.81.htm) Oppositional defiant disorder  [313.82](http://www.icd9data.com/2015/Volume1/290-319/300-316/313/313.82.htm) Identity disorder of childhood or adolescence |
| Psychosis | 290–295.95, 297–298.9 | [290](http://www.icd9data.com/2015/Volume1/290-319/290-294/290/default.htm) Dementias  [291](http://www.icd9data.com/2015/Volume1/290-319/290-294/291/default.htm) Alcohol-induced mental disorders  [292](http://www.icd9data.com/2015/Volume1/290-319/290-294/292/default.htm) Drug-induced mental disorders  [293](http://www.icd9data.com/2015/Volume1/290-319/290-294/293/default.htm) Transient mental disorders due to conditions classified elsewhere  [294](http://www.icd9data.com/2015/Volume1/290-319/290-294/294/default.htm) Persistent mental disorders due to conditions classified elsewhere  [295](http://www.icd9data.com/2015/Volume1/290-319/295-299/295/295.htm) Schizophrenic disorders  [297](http://www.icd9data.com/2015/Volume1/290-319/295-299/297/297.htm) Delusional disorders  [298](http://www.icd9data.com/2015/Volume1/290-319/295-299/298/298.htm) Other nonorganic psychoses |
| Reaction | 308-309.2, 309.22-309.8, 309.82-309.9 | [308](http://www.icd9data.com/2015/Volume1/290-319/300-316/308/308.htm) Acute reaction to stress  [309](http://www.icd9data.com/2015/Volume1/290-319/300-316/309/309.htm) Adjustment reaction  [309.0](http://www.icd9data.com/2015/Volume1/290-319/300-316/309/309.0.htm) Adjustment disorder with depressed mood  [309.1](http://www.icd9data.com/2015/Volume1/290-319/300-316/309/309.1.htm) Prolonged depressive reaction  [309.2](http://www.icd9data.com/2015/Volume1/290-319/300-316/309/309.2.htm) Adjustment reaction with predominant disturbance of other emotions  [309.22](http://www.icd9data.com/2015/Volume1/290-319/300-316/309/309.22.htm) Emancipation disorder of adolescence and early adult life  [309.23](http://www.icd9data.com/2015/Volume1/290-319/300-316/309/309.23.htm) Specific academic or work inhibition  [309.24](http://www.icd9data.com/2015/Volume1/290-319/300-316/309/309.24.htm) Adjustment disorder with anxiety  [309.28](http://www.icd9data.com/2015/Volume1/290-319/300-316/309/309.28.htm) Adjustment disorder with mixed anxiety and depressed mood  [309.29](http://www.icd9data.com/2015/Volume1/290-319/300-316/309/309.29.htm) Other adjustment reactions with predominant disturbance of other emotions  [309.3](http://www.icd9data.com/2015/Volume1/290-319/300-316/309/309.3.htm) Adjustment disorder with disturbance of conduct  [309.4](http://www.icd9data.com/2015/Volume1/290-319/300-316/309/309.4.htm) Adjustment disorder with mixed disturbance of emotions and conduct  [309.8](http://www.icd9data.com/2015/Volume1/290-319/300-316/309/309.8.htm) Other specified adjustment reactions  [309.82](http://www.icd9data.com/2015/Volume1/290-319/300-316/309/309.82.htm) Adjustment reaction with physical symptoms  [309.83](http://www.icd9data.com/2015/Volume1/290-319/300-316/309/309.83.htm) Adjustment reaction with withdrawal  [309.89](http://www.icd9data.com/2015/Volume1/290-319/300-316/309/309.89.htm) Other specified adjustment reactions  [309.9](http://www.icd9data.com/2015/Volume1/290-319/300-316/309/309.9.htm) Unspecified adjustment reaction |
| Substance | 303-305.93 | [303](http://www.icd9data.com/2015/Volume1/290-319/300-316/303/303.htm) Alcohol dependence syndrome  [304](http://www.icd9data.com/2015/Volume1/290-319/300-316/304/default.htm) Drug dependence  [305](http://www.icd9data.com/2015/Volume1/290-319/300-316/305/305.htm) Nondependent abuse of drugs |
| Others |  |  |
| Autism | 299-299.91 | [299](http://www.icd9data.com/2015/Volume1/290-319/295-299/299/299.htm) Pervasive developmental disorders  [299.0](http://www.icd9data.com/2015/Volume1/290-319/295-299/299/299.0.htm) Autistic disorder  [299.1](http://www.icd9data.com/2015/Volume1/290-319/295-299/299/299.1.htm) Childhood disintegrative disorder  [299.8](http://www.icd9data.com/2015/Volume1/290-319/295-299/299/299.8.htm) Other specified pervasive developmental disorders  [299.9](http://www.icd9data.com/2015/Volume1/290-319/295-299/299/299.9.htm) Unspecified pervasive developmental disorder |
| Personality disorders | 301-301.9 | [301](http://www.icd9data.com/2015/Volume1/290-319/300-316/301/301.htm) Personality disorders |
| Sexuality disorders | 302-302.9 | [302](http://www.icd9data.com/2015/Volume1/290-319/300-316/302/default.htm) Sexual and gender identity disorders |
| Eating disorders | 307.1, 307.5-307.53 | [307.1](http://www.icd9data.com/2015/Volume1/290-319/300-316/307/307.1.htm) Anorexia nervosa  [307.5](http://www.icd9data.com/2015/Volume1/290-319/300-316/307/307.5.htm) Other and unspecified disorders of eating  [307.50](http://www.icd9data.com/2015/Volume1/290-319/300-316/307/307.50.htm) Eating disorder, unspecified  [307.51](http://www.icd9data.com/2015/Volume1/290-319/300-316/307/307.51.htm) Bulimia nervosa  [307.52](http://www.icd9data.com/2015/Volume1/290-319/300-316/307/307.52.htm) Pica  [307.53](http://www.icd9data.com/2015/Volume1/290-319/300-316/307/307.53.htm) Rumination disorder |
| Motor disorders | 307.2-307.3 | [307.2](http://www.icd9data.com/2015/Volume1/290-319/300-316/307/307.2.htm) Tics  [307.3](http://www.icd9data.com/2015/Volume1/290-319/300-316/307/307.3.htm) Stereotypic movement disorder |
| Elimination disorders | 307.6, 307.7, 787.6 | [307.6](http://www.icd9data.com/2015/Volume1/290-319/300-316/307/307.6.htm) Enuresis  [307.7](http://www.icd9data.com/2015/Volume1/290-319/300-316/307/307.7.htm) Encopresis  Note: (787.6 not included since it is outside the MH codes), |
| Attachment and nonspecified disorders of infancy | 313.89, 313.9 | [313.89](http://www.icd9data.com/2015/Volume1/290-319/300-316/313/313.89.htm) Other emotional disturbances of childhood or adolescence  [313.9](http://www.icd9data.com/2015/Volume1/290-319/300-316/313/313.9.htm) Unspecified emotional disturbance of childhood or adolescence |
| Childhood, or adolescent ADHD | 314-314.9 | [314](http://www.icd9data.com/2015/Volume1/290-319/300-316/314/314.htm) Hyperkinetic syndrome of childhood |
| Developmental disorder | 315-315.9, 317-319 | [315](http://www.icd9data.com/2015/Volume1/290-319/300-316/315/315.htm) Specific delays in development  [317](http://www.icd9data.com/2015/Volume1/290-319/317-319/317/default.htm) Mild intellectual disabilities  [318](http://www.icd9data.com/2015/Volume1/290-319/317-319/318/default.htm) Other specified intellectual disabilities  [319](http://www.icd9data.com/2015/Volume1/290-319/317-319/319/default.htm) Unspecified intellectual disabilities |
| Miscellaneous | 307-307.0,  307.4-307.49  307.8,  307.9  310-310.9, 316,  306  313 | [307](http://www.icd9data.com/2015/Volume1/290-319/300-316/307/307.htm) Special symptoms or syndromes not elsewhere classified  [307.0](http://www.icd9data.com/2015/Volume1/290-319/300-316/307/307.0.htm) Adult onset fluency disorder  [307.4](http://www.icd9data.com/2015/Volume1/290-319/300-316/307/307.4.htm) Specific disorders of sleep of nonorganic origin  [307.8](http://www.icd9data.com/2015/Volume1/290-319/300-316/307/307.8.htm) Pain disorders related to psychological factors  [307.9](http://www.icd9data.com/2015/Volume1/290-319/300-316/307/307.9.htm) Other and unspecified special symptoms or syndromes, not elsewhere classified  [310](http://www.icd9data.com/2015/Volume1/290-319/300-316/310/310.htm) Specific nonpsychotic mental disorders due to brain damage  [316](http://www.icd9data.com/2015/Volume1/290-319/300-316/316/316.htm) Psychic factors associated with diseases classified elsewhere  [306](http://www.icd9data.com/2015/Volume1/290-319/300-316/306/306.htm) Physiological malfunction arising from mental factors  [313](http://www.icd9data.com/2015/Volume1/290-319/300-316/313/313.htm) Disturbance of emotions specific to childhood and adolescence |
| Suicide/self-inflicted injury | E code 95 | E95 Suicide and self-inflicted injury |

Notes. Minor revisions were made to the Bardach et al. (2014) mental health diagnostic groupings: we removed ICD-9 codes already coded as motor (307.2-307.3) and elimination (307.7) from Mischellaneous; and we included in in Mischellaneous mental health codes not listed elsewhere (i.e., 306 and 313); we added a new Suicide group to include admissions of suicide and self-inflicted injury coded by the external cause of injury code (i.e., E95).

Table S2. Descriptive statistics of mental health-related admissions, separate for hospitals and emergency departments (ED) in New York City, July to August, 2005-2011.

|  | Hospital admissions | | | | ED admissions | | | |
| --- | --- | --- | --- | --- | --- | --- | --- | --- |
|  | 6-11 years  (*n* = 1425) | 12-17 years  (*n* = 5054) | 18-25 years  (*n* = 16,745) | Total  (*n* = 23,251) | 6-11 years  (*n* = 4,816) | 12-17 years  (*n* = 17,152) | 18-25 years  (*n* = 37,763) | Total  (*n* = 59,731) |
| Sex |  |  |  |  |  |  |  |  |
| Female | 449 (30.9%) | 2750 (54.4%) | 6199 (37.0%) | 9398 (40.4%) | 1544 (32.1%) | 9118 (53.160%) | 16107 (42.653%) | 26769 (44.816%) |
| Male | 1003 (69.1%) | 2304 (45.6%) | 10546 (63.0%) | 13853 (59.6%) | 3272 (67.9%) | 8033 (46.834%) | 21655 (57.344%) | 32960 (55.181%) |
| Race/ethnicity |  |  |  |  |  |  |  |  |
| Hispanic | 332 (22.9%) | 1152 (22.8%) | 3418 (20.4%) | 4902 (21.1%) | 1302 (27.0%) | 4628 (27.0%) | 8798 (23.3%) | 14728 (24.7%) |
| NH Black | 668 (46.0%) | 1921 (38.0%) | 5797 (34.6%) | 8386 (36.1%) | 2196 (45.6%) | 6336 (36.9%) | 11890 (31.5%) | 20422 (34.2%) |
| NH Other | 281 (19.4%) | 1205 (23.8%) | 3526 (21.1%) | 5012 (21.6%) | 892 (18.5%) | 3832 (22.3%) | 9386 (24.9%) | 14110 (23.6%) |
| NH White | 138 (9.5%) | 732 (14.5%) | 3911 (23.4%) | 4781 (20.6%) | 402 (8.3%) | 2269 (13.2%) | 7463 (19.8%) | 10134 (17.0%) |
| Unknown/Unreported | 33 (2.3%) | 44 (0.9%) | 93 (0.6%) | 170 (0.7%) | 24 (0.5%) | 87 (0.5%) | 226 (0.6%) | 337 (0.6%) |
| Payment sources |  |  |  |  |  |  |  |  |
| Commercial | 529 (36.4%) | 2598 (51.4%) | 4944 (29.525%) | 8071 (34.712%) | 1757 (36.5%) | 6176 (36.0%) | 9471 (25.1%) | 17404 (29.1%) |
| Medicaid | 860 (59.2%) | 1962 (38.8%) | 8667 (51.759%) | 11489 (49.413%) | 967 (20.1%) | 2804 (16.3%) | 5382 (14.3%) | 9153 (15.3%) |
| Self-pay | 21 (1.4%) | 175 (3.5%) | 1685 (10.063%) | 1881 (8.090%) | 577 (12.0%) | 2501 (14.6%) | 10895 (28.9%) | 13973 (23.4%) |
| Others | 42 (2.9%) | 319 (6.3%) | 1448 (8.647%) | 1809 (7.780%) | 179 (3.7%) | 995 (5.8%) | 2064 (5.5%) | 3238 (5.4%) |
| Unknown/Unreported | - | - | - | - | 1336 (27.7%) | 4676 (27.3%) | 9951 (26.4%) | 15963 (26.7%) |
| Mental health diagnosis |  |  |  |  |  |  |  |  |
| Anxiety | 62 (4.3%) | 120 (2.4%) | 198 (1.2%) | 380 (1.6%) | 470 (9.8%) | 1764 (10.3%) | 5624 (14.9%) | 7858 (13.2%) |
| Bipolar | 77 (5.3%) | 669 (13.2%) | 2588 (15.5%) | 3334 (14.3%) | 124 (2.6%) | 1080 (6.3%) | 2265 (6.0%) | 3469 (5.8%) |
| Depression | 270 (18.6%) | 1520 (30.1%) | 2666 (15.9%) | 4456 (19.2%) | 500 (10.4%) | 3112 (18.1%) | 4992 (13.2%) | 8604 (14.4%) |
| Externalizing | 361 (24.9%) | 841 (16.6%) | 369 (2.2%) | 1571 (6.8%) | 1323 (27.5%) | 3720 (21.7%) | 1082 (2.9%) | 6125 (10.3%) |
| Psychosis | 127 (8.7%) | 678 (13.4%) | 6960 (41.6%) | 7765 (33.4%) | 148 (3.1%) | 799 (4.7%) | 5030 (13.3%) | 5977 (10.0%) |
| Reaction | 54 (3.7%) | 214 (4.2%) | 574 (3.4%) | 842 (3.6%) | 546 (11.3%) | 1572 (9.2%) | 2615 (6.9%) | 4733 (7.9%) |
| Substance | <=10 (0.3%) | 156 (3.1%) | 1775 (10.6%) | 1935 (8.3%) | 13 (0.3%) | 2229 (13.0%) | 12730 (33.7%) | 14972 (25.1%) |
| Suicide/self-inflicted injury | 14 (1.0%) | 490 (9.7%) | 1351 (8.1%) | 1855 (8.0%) | 47 (1.0%) | 789 (4.6%) | 1610 (4.3%) | 2446 (4.1%) |
| Others | 483 (33.3%) | 366 (7.2%) | 264 (1.6%) | 1113 (4.8%) | 1645 (34.2%) | 2087 (12.2%) | 1815 (4.8%) | 5547 (9.3%) |

Notes. Others mental health diagnosis category includes ADHD (n=3144), attachment (n=39), Autism (n=687), Developmental (n=95), eating disorders (n=458), elimination (n=3), others (n=283), motor (n= 57), personality (n=417), sexuality (12), and NA (757) in hospital admissions; and ADHD (n= 14462), attachment (n= 477), Autism (n= 2215), Developmental (n= 1137), eating disorders (n= 277), elimination (n= 21), others (n= 4689), motor (n= 280), personality (n= 1952), sexuality (258), and NA (3510) in ED admissions. Due to data availability, a third of ED patients have empty field in their payment source variable. Other payment sources included worker’s compensation, Medicare, other federal program, blue cross, CHAMPUS, and other non-federal program.

Table S3. Distribution of selected weather variables for all years, June to August, and July to August, 2005-2011, New York City.

| Environmental variables | Mean | Standard deviation | Percentiles | | | | | | |  | Correlations | | | |
| --- | --- | --- | --- | --- | --- | --- | --- | --- | --- | --- | --- | --- | --- | --- |
|  |  |  | Minimum | 25^th^ | 50^th^ | 75^th^ | 95^th^ | 97.5^th^ | Maximum |  | Tmin | Tmax | Tmean | RH |
| *June to August* | | | | | | | | | | | | | | |
| Daily Temperature (ºF) |  |  |  |  |  |  |  |  |  |  |  |  |  |  |
| Tmin | 67.9 | 5.4 | 50.5 | 64.5 | 68.6 | 71.8 | 76.0 | 77.5 | 82.8 |  | - |  |  |  |
| Tmax | 83.7 | 7.0 | 60.5 | 80.0 | 84.0 | 88.5 | 94.8 | 96.5 | 104.8 |  | 0.79 | - |  |  |
| Tmean | 76.1 | 5.8 | 58.8 | 72.8 | 76.3 | 80.0 | 85.0 | 86.2 | 94.0 |  | 0.93 | 0.96 | - |  |
| RH (%) | 65.1 | 12.8 | 31.6 | 55.5 | 65.0 | 74.9 | 85.4 | 88.5 | 92.9 |  | -0.04 | -0.4 | -0.25 | - |
| *July and August* | | | | | | | | | | | | | | |
| Daily Temperature (ºF) |  |  |  |  |  |  |  |  |  |  |  |  |  |  |
| Tmin | 69.6 | 4.6 | 56.5 | 66.8 | 69.8 | 72.8 | 76.8 | 78.3 | 82.8 |  | - |  |  |  |
| Tmax | 85.1 | 6.2 | 60.5 | 81.5 | 84.8 | 89.4 | 95.3 | 97.8 | 104.8 |  | 0.75 | - |  |  |
| Tmean | 77.6 | 5.0 | 58.8 | 74.5 | 77.5 | 81.0 | 85.3 | 87.3 | 94.0 |  | 0.91 | 0.95 | - |  |
| RH (%) | 64.3 | 12.4 | 34.0 | 53.9 | 64.0 | 73.8 | 84.5 | 86.5 | 92.9 |  | 0.05 | -0.39 | -0.22 | - |
| *All year* | | | | | | | | | | | | | | |
| Daily Temperature (ºF) |  |  |  |  |  |  |  |  |  |  |  |  |  |  |
| Tmin | 48.5 | 16.6 | 5.3 | 35.3 | 48.5 | 63.5 | 72.8 | 74.5 | 82.8 |  | - |  |  |  |
| Tmax | 63.4 | 18.2 | 17.8 | 48.8 | 64.3 | 79.5 | 90.0 | 92.3 | 104.8 |  | 0.96 | - |  |  |
| Tmean | 56.2 | 17.2 | 13.8 | 42.3 | 56.8 | 71.8 | 81.0 | 83.3 | 94.0 |  | 0.99 | 0.99 | - |  |
| RH (%) | 62.3 | 15.1 | 19.1 | 50.6 | 61.3 | 74.1 | 87.4 | 89.8 | 96.1 |  | 0.27 | 0.15 | 0.21 | - |

Abbreviations: Tmin = minimum temperature; Tmax= maximum temperature; Tmean = mean temperature; RH = relative humidity. Correlation coefficients were computed using Pearson correlations.

Appendix 1. Model results for separate hospital and emergency departments (ED) admission outcomes.

Figure S1. Cumulative odds ratio (ORs) and 95% confidence intervals of mental health-related admissions, separate for hospitals and emergency departments (ED), associated with daily minimum temperature over lag 0-5 days, June to August.

Table S4. Cumulative odds ratio (ORs) and 95% confidence intervals of mental health-related admissions, separate for hospitals and emergency departments (ED), associated with elevated minimum temperature (95^th^ percentile) relative to the minimum risk temperature.

|  | 6-11 years |  | 12-17 years |  | 18-25 years |  |
| --- | --- | --- | --- | --- | --- | --- |
| Outcome | Minimum  risk  temperature | OR (95% CI) | Minimum  risk  temperature | OR (95% CI) | Minimum  risk  temperature | OR (95% CI) |
| Hospital admissions | 67.9 | **1.29 (1.00, 1.65)** | 65.2 | **1.34 (1.16, 1.54)** | 56.8 | **1.11 (1.00, 1.24)** |
| ED admissions | 69.5 | **1.28 (1.10, 1.49)** | 65.2 | **1.11 (1.03, 1.21)** | 82.7 | 1.12 (0.98, 1.28) |

Note. Bold estimates indicate significant associations with a *p*-value greater than 0.05.

Appendix 2. Model results including relative humidity as a covariate.

Figure S2. Cumulative odds ratio (ORs) and 95% confidence intervals of mental health-related emergency department and hospital admissions associated with daily minimum temperature over lag 0-5 days, June to August, adjusting for relative humidity.

Table S5. Cumulative odds ratio (ORs) and 95% confidence intervals of mental health-related emergency department and hospital admissions associated with elevated minimum temperature (95^th^ percentile) relative to the minimum risk temperature over lag 0-5 days, June to August, adjusting for relative humidity.

|  | 6-11 years |  | 12-17 years |  | 18-25 years |  |
| --- | --- | --- | --- | --- | --- | --- |
| Outcome | Minimum  risk  temperature | OR (95% CI) | Minimum  risk  temperature | OR (95% CI) | Minimum  risk  temperature | OR (95% CI) |
| All admissions | 69.3 | **1.28 (1.13, 1.46)** | 65.2 | **1.17 (1.09, 1.26)** | 58.7 | **1.09 (1.04, 1.15)** |

Note. Bold estimates indicate significant associations with a *p*-value greater than 0.05.

Figure S3. Cumulative odds ratio (ORs) and 95% confidence intervals of mental health-related emergency department and hospital admissions associated with daily minimum temperature over lag 0-5 days, June to August, for sex subgroups.

 Figure S4. Cumulative odds ratio (ORs) and 95% confidence intervals of mental health-related emergency department and hospital admissions associated with daily minimum temperature over lag 0-5 days, June to August, for race subgroups.


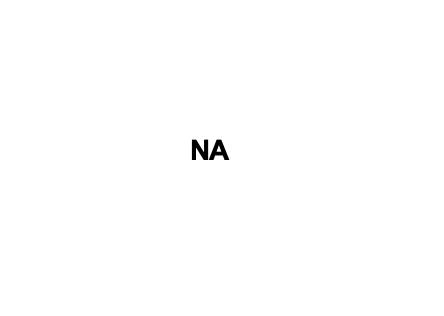


Figure S5. Cumulative odds ratio (ORs) and 95% confidence intervals of mental health-related emergency department and hospital admissions associated with daily minimum temperature over lag 0-5 days, June to August, for payment sources subgroups. We did not present results for small subgroups (less than 500) due to large margins of error.


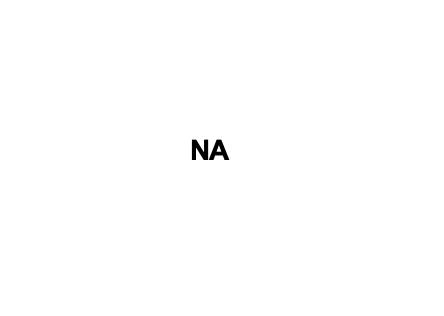


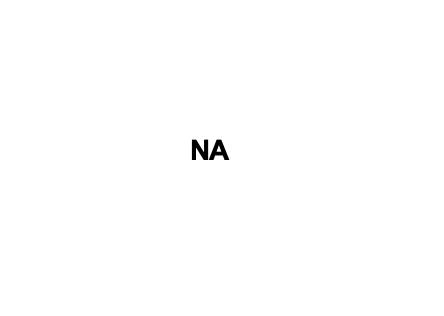


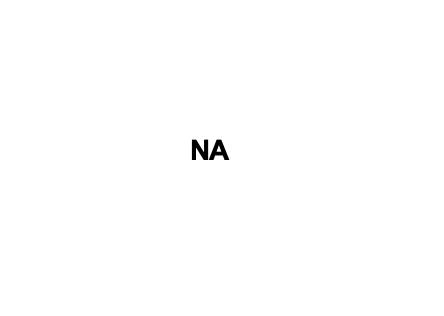


Figure S6. Cumulative odds ratio (ORs) and 95% confidence intervals of mental health-related emergency department and hospital admissions associated with daily minimum temperature over lag 0-5 days, June to August, for mental health diagnostic subgroups. We did not present results for small subgroups (less than 500) due to large margins of error.

Appendix 3. Model results using natural cubic spline with 5 degrees of freedom.

Figure S7. Cumulative odds ratio (ORs) and 95% confidence intervals of mental health-related emergency department and hospital admissions associated with daily minimum temperature over lag 0-5 days, June to August, using natural cubic spline with 5 degrees of freedom.

Table S6. Cumulative odds ratio (ORs) and 95% confidence intervals of mental health-related emergency department and hospital admissions associated with elevated minimum temperature (95^th^ percentile) relative to the minimum risk temperature over lag 0-5 days, June to August, using natural cubic spline with 5 degrees of freedom.

|  | 6-11 years |  | 12-17 years |  | 18-25 years |  |
| --- | --- | --- | --- | --- | --- | --- |
| Outcome | MRT | OR (95% CI) | MRT | OR (95% CI) | MRT | OR (95% CI) |
| All admissions | 69.3 | **1.47 (1.19, 1.81)** | 62.3 | **1.19 (1.09, 1.30)** | 60.3 | **1.08 (1.01, 1.15)** |

Note. MRT is minimum risk temperature. Bold estimates indicate significant associations with a *p*-value greater than 0.05.

Appendix 4. Model results limiting to only July and August.

 Figure S8. Cumulative odds ratio (ORs) and 95% confidence intervals of mental health-related emergency department and hospital admissions associated with daily minimum temperature over lag 0-5 days, July and August.

Table S7. Cumulative odds ratio (ORs) and 95% confidence intervals of mental health-related emergency department and hospital admissions associated with elevated minimum temperature (95^th^ percentile) relative to the minimum risk temperature over lag 0-5 days, July and August.

|  | 6-11 years |  | 12-17 years |  | 18-25 years |  |
| --- | --- | --- | --- | --- | --- | --- |
| Outcome | MRT | OR (95% CI) | MRT | OR (95% CI) | MRT | OR (95% CI) |
| All admissions | 65.2 | **1.44 (1.20, 1.74)** | 56.5 | **1.62 (1.27, 2.08)** | 56.5 | **1.29 (1.11, 1.50)** |

Note. MRT is minimum risk temperature. Bold estimates indicate significant associations with a *p*-value greater than 0.05.

**Table S8. Heterogeneity test of odds ratio (ORs) of the association between mental health-related emergency department and hospital admissions with elevated minimum temperature (95^th^ percentile) relative to the minimum risk temperature (MRT), by sex, race, payment sources, and mental health diagnosis subgroups.**

|  | 6-11 years | | | 12-17 years | | | 18-25 years | | |
| --- | --- | --- | --- | --- | --- | --- | --- | --- | --- |
|  | MRT | OR (95% CI) | Q^a^ | MRT | OR (95% CI) | Q^a^ | MRT | OR (95% CI) | Q ^a^ |
| All admissions | 69.2 | **1.28 (1.13, 1.46)** | - | 65.2 | **1.17 (1.09, 1.25)** | - | 58.7 | **1.09 (1.04, 1.15)** | **-** |
| Sex |  |  |  |  |  |  |  |  |  |
| Female | **68.2** | **1.34 (1.06, 1.71)** | - | **64.4** | **1.26 (1.14, 1.39)** | - | 54.8 | 1.12 (0.99, 1.27) | - |
| Male | **69.5** | **1.28 (1.09, 1.52)** | 0.82 | **69.0** | **1.11 (1.00, 1.22)** | 0.07 | **60.3** | **1.07 (1.00, 1.14)** | 0.52 |
| Race |  |  |  |  |  |  |  |  |  |
| Hispanic | 63.3 | 1.37 (0.99, 1.90) | - | 66.4 | 1.14 (0.97, 1.33) | - | 50.5 | 1.17 (0.87, 1.58) | - |
| Non-Hispanic Black | 69.9 | **1.43 (1.12, 1.83)** | 0.90 | 64.1 | **1.21 (1.06, 1.39)** | 0.57 | 50.5 | 1.13 (0.88, 1.44) | 0.86 |
| Non-Hispanic White | 70.6 | 1.33 (0.78, 2.28) | 0.92 | 66.7 | 1.19 (0.97, 1.47) | 0.75 | 82.7 | 1.20 (0.97, 1.49) | 0.89 |
| Non-Hispanic Other | 69.7 | **1.60 (1.12, 2.27)** | 0.64 | 68.6 | **1.26 (1.07, 1.49)** | 0.39 | 62.3 | **1.12 (1.01, 1.25)** | 0.79 |
| Payment source |  |  |  |  |  |  |  |  |  |
| Commercial | 70.2 | 1.19 (0.88, 1.60) | - | 64.3 | **1.25 (1.08, 1.44)** | - | 50.5 | 1.29 (0.98, 1.71) | - |
| Medicaid | 66.9 | **1.48 (1.09, 2.02)** | 0.25 | 63.8 | 1.11 (0.92, 1.34) | 0.32 | 62.8 | **1.20 (1.08, 1.33)** | 0.64 |
| Self-pay | 70.4 | 1.71 (0.90, 3.27) | 0.17 | 63.8 | 1.21 (0.91, 1.60) | 0.84 | 82.7 | **1.33 (1.06, 1.67)** | 0.87 |
| Others | - | - |  | 71.4 | 1.15 (0.81, 1.63) | 0.66 | 50.5 | 1.43 (0.80, 2.56) | 0.75 |
| Mental health diagnosis |  |  |  |  |  |  |  |  |  |
| Anxiety disorders | 50.5 | 1.66 (0.45, 6.12) | - | 63.1 | **1.42 (1.08, 1.87)** | - | 82.7 | 1.15 (0.85, 1.57) | - |
| Bipolar disorder | - | - | - | 69.6 | **1.39 (1.06, 1.83)** | 0.91 | 50.5 | 1.37 (0.85, 2.22) | 0.55 |
| Depression | 69.5 | 1.31 (0.83, 2.07) | 0.72 | 66.8 | 1.13 (0.94, 1.36) | 0.18 | 82.7 | 1.26 (0.96, 1.66) | 0.67 |
| Externalizing disorders | 69.0 | 1.20 (0.86, 1.68) | 0.64 | 65.6 | 1.08 (0.90, 1.30) | 0.11 | 50.5 | 1.97 (0.83, 4.68) | 0.25 |
| Psychosis | - | - | - | 57.5 | 1.34 (0.93, 1.94) | 0.81 | 65.1 | **1.14 (1.03, 1.27)** | 0.96 |
| Reaction disorders | 69.7 | **2.21 (1.22, 3.99)** | 0.70 | 63.8 | 1.23 (0.90, 1.68) | 0.50 | 58.5 | **1.32 (1.04, 1.67)** | 0.49 |
| Substance abuse | - | - | - | 82.7 | 1.06 (0.66, 1.72) | 0.30 | 50.5 | 1.20 (0.91, 1.60) | 0.84 |
| Suicide/self-inflicted injury | - | - | - | 65.8 | 1.19 (0.85, 1.67) | 0.43 | 50.5 | 1.61 (0.87, 2.98) | 0.34 |

Notes. We did not present results for small subgroups (less than 500) due to large margins of error. Bold estimates indicate significant associations with a *p*-value greater than 0.05. ^a^ Statistical evidence for effect modification was assessed by estimating the degree of heterogeneity between subgroup-specific ORs in pairwise comparisons (Kaufman and MacLehose 2013). P-value associated with Cochran’s Q test statistics are presented. Referent groups for each stratum were female sex, Hispanic, commercial, and anxiety disorders.
